# Sequential Enzymatic Conversion of α‐Angelica Lactone to γ‐Valerolactone through Hydride‐Independent C=C Bond Isomerization

**DOI:** 10.1002/cssc.201601363

**Published:** 2016-11-25

**Authors:** Nikolaus G. Turrini, Elisabeth Eger, Tamara C. Reiter, Kurt Faber, Mélanie Hall

**Affiliations:** ^1^ Department of Chemistry University of Graz Heinrichstrasse 28 8010 Graz Austria; ^2^ ACIB GmbH, Department of Chemistry University of Graz Heinrichstrasse 28 8010 Graz Austria

**Keywords:** biobased chemicals, biocatalysis, ene-reductases, isomerization, α-angelica lactone

## Abstract

A case of hydride‐independent reaction catalyzed by flavin‐dependent ene‐reductases from the Old Yellow Enzyme (OYE) family was identified. α‐Angelica lactone was isomerized to the conjugated β‐isomer in a nicotinamide‐free and hydride‐independent process. The catalytic cycle of C=C bond isomerization appears to be flavin‐independent and to rely solely on a deprotonation–reprotonation sequence through acid–base catalysis. Key residues in the enzyme active site were mutated and provided insight on important mechanistic features. The isomerization of α‐angelica lactone by OYE2 in aqueous buffer furnished 6.3 mm β‐isomer in 15 min at 30 °C. In presence of nicotinamide adenine dinucleotide (NADH), the latter could be further reduced to γ‐valerolactone. This enzymatic tool was successfully applied on semi‐preparative scale and constitutes a sustainable process for the valorization of platform chemicals from renewable resources.

Ene‐reductases from the Old Yellow Enzyme (OYE) family are flavin‐dependent enzymes that were well investigated for their ability to catalyze the asymmetric reduction of activated C=C bonds at the expense of nicotinamide cofactor.^1^ In the first (reductive) half‐reaction, flavin mononucleotide (FMN) is reduced by externally added nicotinamide adenine dinucleotide (phosphate) [NAD(P)H]. The reduced FMN, in turn, can transfer a hydride onto the β‐carbon of a C=C bond, which is activated by an electron‐withdrawing group (oxidative half reaction).^2^ In special cases, mostly on cyclic compounds such as α,β‐unsaturated cycloalkenones or lactones, distinct unusual catalytic behaviors of OYE homologues were observed: 1) Nicotinamide‐free dismutation catalyzed by OYE1 was identified using cyclohexen‐2‐one and 3‐oxodecalin‐4‐ene, in which one substrate molecule is first dehydrogenated by oxidized flavin, followed by reduction of a second molecule by the reduced flavin (Scheme 1 A);^3^ this intermolecular reaction was extended to a broad variety of substrates and OYE homologues.^4^ 2) Redox‐neutral C=C bond isomerization of α‐methylene‐γ‐butyrolactone to thermodynamically more stable 3‐methylfuran‐2(5 *H*)‐one was observed with OYE2 (Scheme 1 B); this process requires only catalytic amounts of NADH to activate the flavin by means of reduction, thereby triggering intermolecular hydride transfer from *endo*‐Cβ onto *exo*‐Cβ through FMN.^5^ In both cases, the flavin acts as hydride shuttle between two substrate molecules. In contrast to these redox‐neutral isomerization reactions, C=C reduction of racemic γ‐substituted α,β‐unsaturated lactones proceeds through kinetic resolution, and dynamic kinetic resolution of α,γ‐disubstituted analogues was achieved with the OYE homologue nicotinamide‐dependent cyclohexenone reductase (NCR) from *Zymomonas mobilis*.^6^ The reduction reactions resemble a 1,4‐Michael‐type addition and require a stoichiometric amount of nicotinamide cofactor (Scheme 1 C). Until now, the catalytic cycle of ene‐reductases was reported to solely rely on hydride transfer using the reduced flavin, and no hydride‐independent catalysis was reported.

**Scheme 1:**
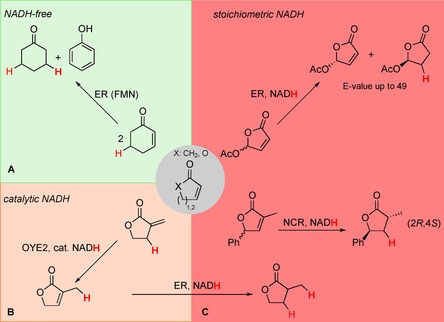
Versatile catalytic behavior of ene‐reductases (ER) on α,β‐unsaturated cycloalkenones and lactones. A) NADH‐independent intermolecular hydrogen transfer; B) NADH‐catalyzed intermolecular hydrogen transfer; C) stoichiometric NADH‐dependent C=C reduction. E‐value: enantiomeric ratio.

Levulinic acid derived from cellulosic feedstocks represents a major biobased platform chemical.^7^ Upon dehydration, α‐angelica lactone is formed, which can be isomerized to β‐angelica lactone or reduced to γ‐valerolactone, two versatile building blocks with broad applications (e.g., precursors of biobased polymers and natural products, solvents, fuel additives).^8^ Because OYEs can only reduce activated C=C bonds, α‐angelica lactone does not appear to be a suitable substrate, but the C=C isomerization activity of some OYE homologues on α,β‐unsaturated cycloalkenones and lactones encouraged us to investigate the reactivity of α‐angelica lactone (**1 a**) with a panel of ene‐reductases (OYE2, OYE3, NCR, YqjM, OPR1, OPR3, EBP1, XenA).

The initial setup consisted of substrate solution (10 mm) in Tris‐HCl [tris(hydroxymethyl)aminomethane] buffer (pH 7.5, 50 mm), excess of NADH (1.5 equiv) and enzyme (100 μg mL^‐1^, approximately 2.2 μm). Upon extraction of the aqueous solution with ethyl acetate after 24 h and subsequent GC–MS analysis, γ‐valerolactone (**1 c**) was identified as single product from the reaction with OYE2 from *Saccharomyces cerevisiae* whereas no starting material could be recovered, indicating that OYE2 was able to convert α‐angelica lactone. Other ene‐reductases appeared inactive. This result was surprising because reduction of C=C bonds on analogous compounds was only reported on enone derivatives so far (i.e., 1,4‐addition on activated C=C bonds). By analogy with the isomerization reaction of α‐methylene‐γ‐butyrolactone catalyzed by OYE2,^5^ which requires catalytic amounts of nicotinamide, the experiment was repeated with lower cofactor concentration. Upon reducing NADH concentration to substoichiometric amounts, isomerized product β‐angelica lactone (**1 b**) could be detected, whereas consistently less saturated product **1 c** was produced (Table 1). At 10 μm NADH (0.1 mol %), no reduction product was detected whereas **1 b** was formed in 4 mm. The overall low recovery was attributed to reversible hydrolytic ring opening of α‐angelica lactone,^9^ which appeared much more unstable than the β‐isomer **1 b** in aqueous solution (data not shown). The product distribution depended highly on NADH concentration, and clearly showed that **1 c** arises from reduction of **1 b**, which itself derives from isomerization of **1 a** (Scheme 2). In addition, the reaction of **1 b** (synthesized according to published procedure,^10^ Supporting Information) in presence of OYE2 and excess of NADH led to formation of **1 c** as sole product. In both cases (reaction starting from **1 a** or **1 b**), all compounds were racemic. Reduction of *rac*‐5‐oxo‐2,5‐dihydrofuran‐2‐yl acetate, chemically analogous to **1 b**, proceeded through kinetic resolution with a handful of ene‐reductases, but OYE2 afforded the racemic product.^6^

**Table 1:** Product distribution after reaction of **1 a** (10 mm) with OYE2 at varying NADH concentrations.^[a]^

Entry	Concentration [mm]			
	NADH	**1 a**	**1 b**	**1 c**
1	10	n.d.	0.19	2.03
2	5	0.13	0.60	2.19
3	2	0.57	2.37	0.99
4	0.5	0.82	3.38	0.23
5	0.1	1.05	4.25	0.03
6	0.05	0.83	3.70	0.04
7	0.01	0.93	4.03	n.d.
8	0^[b]^	1.50	6.30	n.d.

[a] Tris‐HCl buffer (50 mm, pH 7.5), 30 °C, 120 rpm, 3 h reaction time; n.d.=not detected. [b] Reaction time: 15 min.

**Scheme 2:**
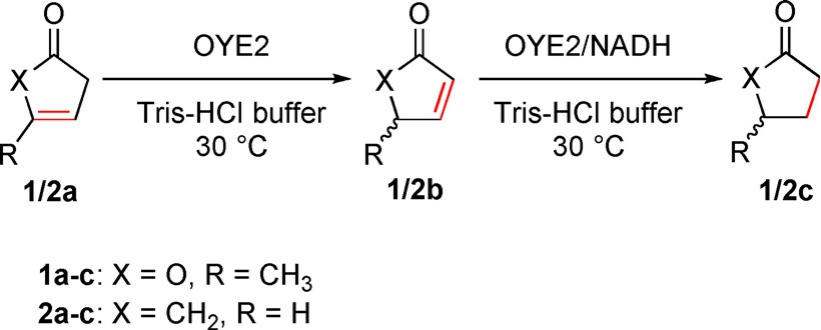
Sequential biotransformation of unactivated substrates **1 a** and **2 a** by OYE2 from *Saccharomyces cerevisiae* to **1 c** and **2 c**, respectively.

Finally, the reaction was performed in absence of NADH. Almost full consumption of **1 a** (85 %) could be observed within 15 min, leading to the formation of 6.3 mm **1 b**. Among all OYE homologues tested in absence of NADH, only OYE3 showed significant activity in the nicotinamide‐free C=C bond isomerization (35 % conversion in 24 h, Table 2, entry 3). Clearly, this enzymatic isomerization is distinct from the isomerization of α‐methylene‐γ‐butyrolactone by OYE2, which requires catalytic amounts of NADH cofactor to initiate hydride transfer.^5^ By analogy with the isomerization mechanism of Δ^5^‐3‐ketosteroid isomerase on steroids,^11^ the isomerization of the unactivated C=C bond in α‐angelica lactone by OYE2 most likely relies on acid–base catalysis and is hydride independent. FMN is naturally occurring in oxidized form in OYE homologues and thus cannot initiate hydride addition.

**Table 2:** Nicotinamide‐free isomerization of **1 a** and **2 a** to **1 b** and **2 b** catalyzed by OYEs.^[a]^

Entry	Substrate	Protein	Concentration [μm]	Conv. [%]
1	**1 a**	OYE2‐wt	2.8	45
2	**1 a**	OYE2‐wt^[b]^	2.8	13
3	**1 a**	OYE3‐wt^[c]^	2.8	35
4	**1 a**	OYE2‐H192A	2.8	40
5	**1 a**	OYE2‐Y197F	2.8	<1
6	**1 a**	OYE2‐Y197F^[d]^	741	30
7	**1 a**	BSA	600	<1
8	**1 a**	OYE2 denatured^[e]^	2.8	<1
9	**2 a**	OYE2‐wt	2.8	49^[f]^
10	**2 a**	OYE2‐H192A	2.8	25
11	**2 a**	OYE2‐Y197F	2.8	15^[g]^

[a] Tris‐HCl buffer (50 mm, pH 7.5), 2 h reaction time; wt=wild type. [b] pH 6. [c] 24 h reaction time. [d] Corresponds to TTN of approximately 4 (3 mm **1 b**). [e] Denaturation procedure: OYE2 sample was heated to 95 °C for 10 min, allowed to cool down, and used in the reaction as such. [f] No trace of **2 a**. [g] Control (no enzyme) also showed 15 % of **2 b**.

To identify possible catalytic residues functioning as acid and/or base, a mutational study was designed based on the crystal structure of highly homologous OYE1 from *Saccharomyces pastorianus* (92 % identity and 95 % similarity).^12^ Two key positions were targeted: 1) His192 (p*K*a≈6) is one of the two residues involved in the binding of the substrate carbonyl through hydrogen bonds and could act as a base using its unprotonated nitrogen atom in the deprotonation of an acidic α‐H of **1 a**;^2^ 2) Tyr197 (p*K*a≈9.1^13^) is the proton donor for the α‐C in the reduction reaction and may function as proton donor in the isomerization (Scheme 3).

**Scheme 3:**
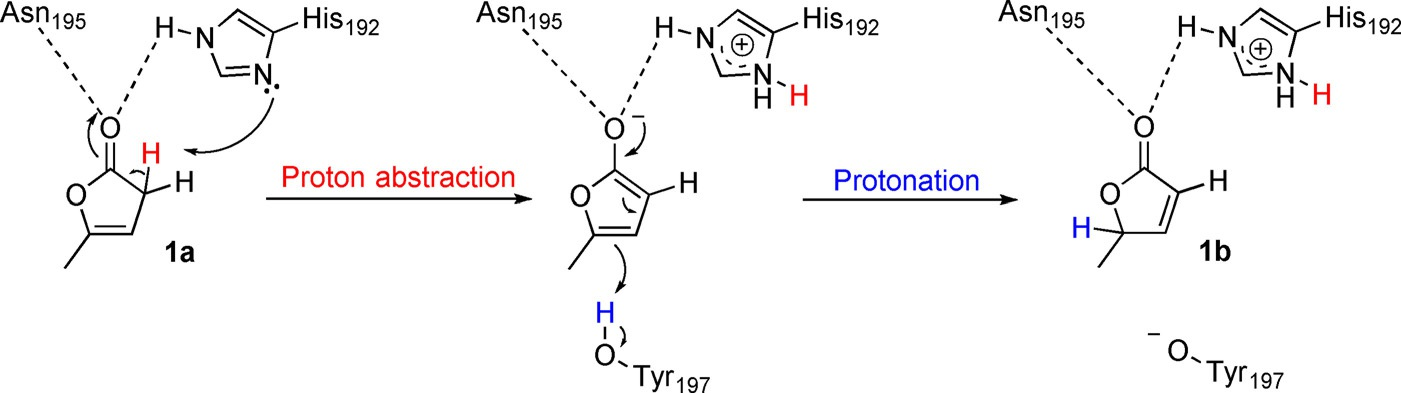
Initial proposed mechanism for the isomerization of α‐angelica lactone by OYE2. Return to histidine (His) and tyrosine (Tyr) initial states is solvent mediated (buffer pH 7.5). Asn=asparagine.

Tyr197 was mutated to phenylalanine (Phe) to conserve similar steric properties but remove the hydrogen‐donor capacity, whereas His192 was mutated to alanine (Ala) to eliminate both H‐bonding capacity and basic character. Both single variants were first evaluated in the reduction of *N*‐phenyl‐2‐methyl‐ maleimide. Successful reduction in the presence of NADH confirmed that both variants were still able to bind the substrate (Supporting Information). OYE2‐H192A could catalyze the isomerization of **1 a** to **1 b** in absence of NADH (Table 2, entry 4), albeit with slightly decreased activity, going in line with lower reduction activity and expected reduced binding affinity resulting from the change from two binding residues (H192/N195) to only one.^14^ In contrast, OYE2‐Y197F at first showed no activity under standard reaction conditions (2.2 μm enzyme, Table 2, entry 5). When the concentration was increased to 741 μm, 3 mm of **1 b** were obtained (Table 2, entry 6), indicating that the catalytic activity of the variant was severely affected [total turnover number (TTN) ≈4]. A control reaction in presence of elevated bovine serum albumin (BSA) concentration or denatured OYE2 resulted in no product (Table 2, entries 7 and 8), confirming that the low turnover did not result from nonspecific amino‐acid catalysis.

Taken together, the data suggest that His192 is not the catalytic base, although the role of Tyr197 remains unclear. Based on the proposed mechanism (Scheme 3), an initial deprotonation step would generate an aromatic anion as intermediate, which resembles a phenolate. Phenols are notorious inhibitors of OYEs owing to strong binding between phenolate and oxidized FMN.^15^ Similarly, the suggested anionic aromatic intermediate would strongly interact with oxidized FMN and, in absence of a proton donor in OYE2‐Y197F, cannot (or only slowly) leave the active site, thereby preventing product formation. In contrast, the reducing activity of variants lacking the catalytic Tyr is not strongly affected (Supporting Information) because the nonaromatic intermediate is easily released and further protonated by the solvent (water).2a The significantly lower conversion level at pH 6 (Table 2, entry 2) strongly supports the involvement of a base in the deprotonation step. Although the reaction at higher pH values (8–9) led to formation of **1 b**, conversion levels could not be determined owing to the instability of **1 a** in basic medium (data not shown). Elucidation of the (yet unsolved) structure of OYE2 will enable identification of additional point mutations for further mechanistic studies. Interestingly, cases of flavin acting as acid/base were reported, but all require a reduced flavin.^16^

The reaction was extended to 3‐cyclopenten‐1‐one (**2 a**), which was isomerized to 2‐cyclopenten‐1‐one (**2 b**) in a nicotinamide‐independent manner (Table 2, entry 9). Similar to **1 a**, the conversion level with OYE2‐H192A was affected by the mutation and OYE2‐Y197F was inactive (Table 2, entries 10 and 11). Finally, the reaction was scaled up to demonstrate the applicability of the cofactor‐free enzymatic isomerization of α‐angelica lactone to its β‐isomer. **1 a** (55 mg, 25 mm) was converted by OYE2 (0.03 mol %) in Tris‐HCl buffer at 30 °C for 45 min and allowed isolation of 18 mg **1 b** (32 % overall yield, 98 % purity, Experimental Section).

The catalytic activity of ene‐reductases from the Old Yellow Enzyme (OYE) family of flavoproteins was so far restricted to flavin‐ and hydride‐dependent processes in which reduction of flavin mononucleotide (FMN) by an external hydride‐cofactor is the initial step required for activity. Asymmetric stoichiometric nicotinamide‐dependent reduction is broadly applicable to activated α,β‐unsaturated alkenes and includes (dynamic) kinetic resolution of γ‐substituted substrates. Catalytic nicotinamide‐dependent isomerization was observed on α‐methylene‐γ‐butyrolactone, and nicotinamide‐independent disproportionation of enones relies on reduced FMN as hydride shuttle between two substrates molecules. In contrast, the hydride‐ and nicotinamide‐independent isomerization of unactivated C=C bonds reported here represents a new catalytic activity of ene‐reductases. The isomerization of α‐angelica lactone to its β‐isomer by OYE2 enzyme was successfully performed on a preparative scale, and the possibility of further reduction to γ‐valerolactone in a cascade was demonstrated.

## Experimental Section

### Standard procedure for bioreduction reactions

To a solution of Tris‐HCl buffer (800 μL, 50 mm, pH 7.5) containing the substrate (10 mm) and the cofactor (concentration as indicated in Table 1) was added an aliquot of the respective enzymes (final protein concentration 100 μg mL^−1^, approximately 2.2 μm, unless denoted otherwise). All samples were run in duplicates, and control experiments were included in every screening (no enzyme). Substrates were added from a 0.5 m stock solution in ethanol to overcome solubility problems (final concentration of ethanol 2 %). The mixture was shaken at 30 °C and 120 rpm (reaction time as indicated in the text and Tables 1 and 2). Afterwards, the reaction was extracted with ethyl acetate (2×500 μL), and the combined organic layers were dried over Na_2_SO_4_ and transferred into GC vials. Products were identified by comparison with authentic reference material. Limonene (10 mm) was used as internal standard.

### Procedure for scale‐up

To a solution of **1 a** (55.7 mg, 0.57 mmol, 25 mm) in Tris‐HCl buffer (50 mm, pH 7.5) was added an aliquot of OYE2 (2.2 μm). The reaction mixture was incubated at 30 °C and 120 rpm. Analysis of the reaction (GC–MS) after 15 min reaction time showed fast isomerization (**1 b**/**1 a**=90:10). Two aliquots of OYE2 (4.4 μm) were then added (total 0.03 mol %) and the reaction was further incubated for additional 30 min. Extraction was performed with ethyl acetate (3×15 mL), and the combined organic layers were dried over Na_2_SO_4_. Evaporation of the solvent yielded 18.1 mg **1 b** in 32 % overall yield (98 % purity, Supporting Information).

## Supporting information

As a service to our authors and readers, this journal provides supporting information supplied by the authors. Such materials are peer reviewed and may be re‐organized for online delivery, but are not copy‐edited or typeset. Technical support issues arising from supporting information (other than missing files) should be addressed to the authors.

Supplementary
