# Supplementary material for: Sequential Enzymatic Conversion of α‐Angelica Lactone to γ‐Valerolactone through Hydride‐Independent C=C Bond Isomerization
Source: ChemSusChem. 2016 Nov 25;9(24):3393–6. doi: 10.1002/cssc.201601363 (PMC5574032; doi:10.1002/cssc.201601363)
Supplement: Supplementary file 1 — Supplementary [file CSSC-9-3393-s001.pdf]

CHEMISTRY & SUSTAINABILITY
CHEMSUSCHEM
ENERGY & MATERIALS

Supporting Information

Sequential Enzymatic Conversion of α-Angelica Lactone to γ-Valerolactone through Hydride-Independent C=C Bond Isomerization

Nikolaus G. Turrini,[a] Elisabeth Eger,[a] Tamara C. Reiter,[a, b] Kurt Faber,[a] and Mélanie Hall*[a]

cssc_201601363_sm_miscellaneous_information.pdf

Experimental Section

General remarks

GC-MS analyses were performed on a HP Agilent Technologies 6890 Series GC system equipped with a 5973 mass selective detector, a 7683 Series injector and an Agilent HP-5 MSI column, (30 m, 0.25 mm inner diameter, 0.25 µm film thickness). GC-FID analyses were performed using a HP Agilent Technologies 7890A GC system equipped with a FID detector and a 7693 Autosampler or a 7683B Injector in combination with a 7683 Series Autosampler (columns *vide infra*). NMR spectra were measured on a Bruker Avance III 300 MHz NMR spectrometer. Chemical shifts are reported relative to TMS (δ = 0.00 ppm); coupling constants J are given in Hz. All enzymes were overexpressed and purified as reported[1] and used as purified protein (>90% purity). **1a,** *rac*-**1c** and (*S*)-**1c** were obtained from Sigma-Aldrich.

Analytical Methods

Synthesis of reference material (1b)

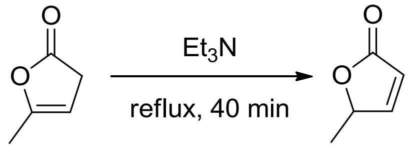

1a **1b**

35 g (350 mmol) of α-angelica lactone (**1a**) were placed in a 100 mL round bottom flask, 313 µL (2.23 mmol) Et_3N were added and the reaction was stirred at reflux for 40 minutes. Afterwards, the reaction was allowed to cool to room temperature. Purification was performed *via* column chromatography (silica gel, eluent: hexanes/EtOAc 3:2). Yield: 9.61 g (97 mmol, 28%) of **1b**.

NMR **1b** (in accordance with literature[2]):

^{1}H NMR (300 MHz, $CDCl_3$) δ 7.45 (dd, J = 5.7, 1.5 Hz, 1H), 6.06 (dd, J = 5.7, 1.9 Hz, 1H), 5.19 – 5.03 (m, 1H), 1.42 (d, J = 6.9 Hz, 3H). ^{13}C NMR (75 MHz, $CDCl_3$) δ 173.17, 157.55, 121.24, 79.72, 18.86.

Determination of conversions

Conversions of the substrates were determined by either GC-FID or GC/MS. For GC/MS, the temperature program was: 40 °C, hold for 2 min, 10 °C/min to 180 °C, hold for 1 min. Retention times: **1a** 6.6 min, **1b** 7.8 min, **1c** 8.10 min, **2a** 4.4 min, **2b** 5.8 min. For achiral GC-FID measurements, an Agilent J&W HP-5 column (30 m x 0.32 mm ID, 0.25 µm film thickness) was used. Temperature program: 40 °C, hold for 2

min, 10 °C/min to 180 °C, hold for 1 min. Retention times: **1a** 4.7 min, **1b** 5.8 min, **1c** 6.1 min, **2a** 3.0 min, **2b** 4.2 min.

GC/MS traces

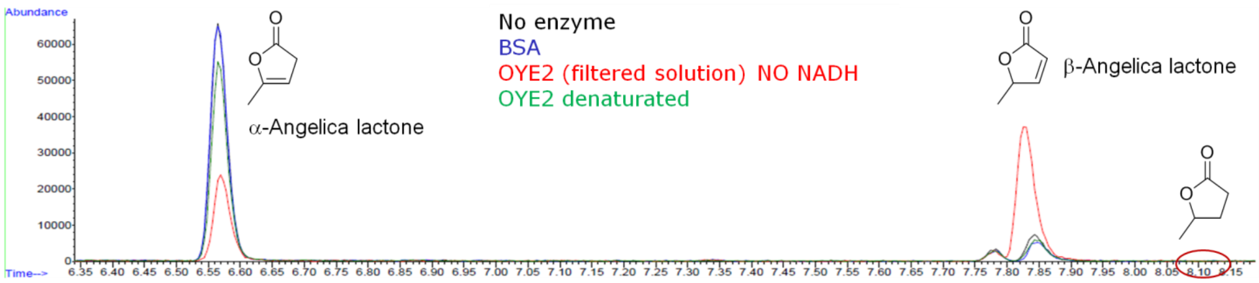

Scale-up

The scale up was performed as described in the experimental section. Upon evaporation of the solvent, GC-MS analysis of the recovered product (18.1 mg) indicated a purity of 98% for β-isomer **1b**.

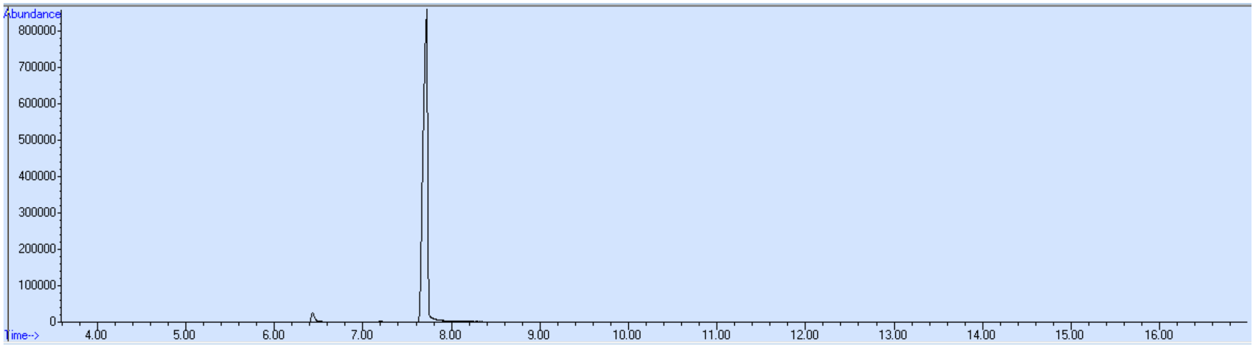

Determination of enantiomeric excess

Optical purity of the products was determined by GC-FID measurements by comparison with authentic reference material. A Chirasil Chiraldex DEX-CB column (25 m, 0.32 mm ID, 0.25 µm film thickness) was used. Temperature program: 60 °C, 5 °C/min. to 85 °C, hold for 5 min., 20 °C/min. to 180 °C. Retention times: (*R/S*)-**1b** 5.5 min, (*S/R*)-**1b** 5.9 min, (*R*)-**1c** 5.4 min, (*S*)-**1c** 5.8 min.

Cloning of OYE2

The synthetic gene for OYE2 was ordered (Thermo Fisher Scientific) and codon-optimized for *E. coli*.

ATGCACCATTTGTTAAGGACTTTAAGCCACAAGCTTTGGGTGACACCAACTTATTCAAACCAATCAAAATTGGTAACAATGAACTT
CTACACCGTGCTGTCATTCCTCCATTGACTAGAATGAGAGCCCAACATCCAGGTAATATTCCAAACAGAGACTGGGCCGTTGAATA
CTACGCTCAACGTGCTCAAAGACCAGGAACCTTGATTATCACTGAAGGTACCTTTCCCTCTCCACAATCTGGGGGTTACGACAATG
CTCCAGGTATCTGGTCCGAAGAACAAATTAAAGAATGGACCAAGATTTTCAAGGCTATTCATGAGAATAAATCGTTCGCATGGGTC
CAATTATGGGTTCTAGGTTGGGCTGCTTTCCCAGACACCCTTGCTAGGGATGGTTTGCGTTACGACTCCGCTTCTGACAACGTGTAT
ATGAATGCAGAACAAGAAGAAAAGGCTAAGAAGGCTAACAACCCACAACACAGTATAACAAAGGATGAAATTAAGCAATACGTC
AAAGAATACGTCCAAGCTGCCAAAAACTCCATTGCTGCTGGTGCCGATGGTGTTGAAATCCACAGCGCTAACGGTTACTTGTTGAA

CCAGTTCTTGGACCCACACTCCAATAACAGAACCGATGAGTATGGTGGATCCATCGAAAACAGAGCCCGTTTCACCTTGGAAGTG
GTTGATGCAGTTGTCGATGCTATTGGCCCTGAAAAAGTCGGTTTGAGATTGTCTCCATATGGTGTCTTCAACAGTATGTCTGGTGGT
GCTGAAACCGGTATTGTTGCTCAATATGCTTATGTCTTAGGTGAACTAGAAAGAAGAGCTAAAGCTGGCAAGCGTTTGGCTTTCGT
CCATCTAGTTGAACCTCGTGTCACCAACCCATTTTTAACTGAAGGTGAAGGTGAATACAATGGAGGTAGCAACAAATTTGCTTATT
CTATCTGGAAGGGCCCAATTATTAGAGCTGGTAACTTTGCTCTGCACCCAGAAGTTGTCAGAGAAGAGGTGAAGGATCCTAGAAC
ATTGATCGGTTACGGTAGATTTTTTATCTCTAATCCAGATTTGGTTGATCGTTTGGAAAAAGGGTTACCATTAAACAAATATGACAG
AGACACTTTCTACAAAATGTCAGCTGAGGGATACATTGACTACCCTACGTACGAAGAAGCTCTAAAACTCGGTTGGGACAAAAAT
TAA

The synthetic gene (plasmid) was cloned into pET28a (+)-vector. Restriction (*Nco*I and *Xho*I) was performed in sterile PCR-tubes at 37 °C for 2 hours. Successful digestion was confirmed by Agarose-gel (1% Agarose in TAE-buffer, 90 V). The bands were excised and DNA was extracted using the QIAquick gel extraction kit. DNA concentrations were determined on the photometer. For ligation, 33 ng (5 µL) of vector and 31.5 ng (7 µL) of OYE2-DNA template were added into PCR-tubes containing 2 µL buffer, 1 µL T4-ligase and 5 µL H_2O. The reaction was left at room temperature for 1 h and then placed in the cold room overnight. On the next day, a transformation into NEB5α was performed. Six ONCs were prepared and the plasmid was isolated the following day. Sequencing showed successful incorporation of the proper plasmid into the vector. Furthermore, Agarose-gel confirmed the insertion of the desired DNA-fragment.

Site-directed Mutagenesis

Primers that included the desired mutations were designed using the sequence of codon-optimized (*E. coli*) OYE2-gene (Table S1). All mutants were generated using an Agilent QuikChange II XL Site-Directed Mutagenesis Kit. The obtained plasmids were transformed into XL10-Gold Ultracompetent Cells according to the manufacturer’s manual. The correct introduction on mutations was confirmed by sequencing.

Table S1: Primers used for mutation studies (triplet encoding the new amino acid in bold letters)

Variant	Primers
OYE2-Y197A_fw	ATAGCGCAAATGGT**GCG**CTGCTGAACCAGTTT
OYE2-Y197A_rv	AAACTGGTTCAGCAG**CGC**ACCATTTGCGCTAT
OYE2-Y197F_fw	ATAGCGCAAATGGT**TTC**CTGCTGAACCAGTTT
OYE2-Y197F_rv	AAACTGGTTCAGCAG**GAA**ACCATTTGCGCTAT
OYE2-H192A_fw	GATGGTGTTGAAATT**GCT**AGCGCAAATGGTTATC
OYE2-H192A_rv	GATAACCATTTGCGCT**AGC**AATTTCAACACCATC

Transformation of plasmids

To 100 µL of cell suspension in 1.5 mL Eppendorf tubes, 10 µL of plasmid were added and incubated on ice for 30 min. The samples were then placed in a thermoshaker 42 °C for 10 s [BL21(DE3)] and 30 s (NEB5α) respectively. Afterwards, 250 µL of LB-medium were added and the cells were incubated in the

thermomixer for 1 h at 37 °C and 300 rpm. 100 µL of the resulting product were plated on kanamycin-containing agar plates.

Overexpression of OYE2 and variants

Colonies obtained from the transformation step were used to prepare overnight cultures (ONCs; 10 mL LB-medium, 50 µg/mL kanamycin) of all mutants. They were placed in the shaker overnight (37 °C, 120 rpm) and used on the next day to inoculate the main cultures (330 mL LB-medium per shaking flask; 3.3 mL of the ONCs, 50 µg/mL kanamycin). The main cultures were placed in the shaker at 37 °C and 140 rpm until an OD_{600} of 0.6 was reached. Then, IPTG (0.2 mM) was used for induction. The cultures were again placed in the shaker overnight at 37 °C and 140 rpm. On the next day, the cells were harvested by centrifugation (4000 rpm, 20 min, 4 °C) and the supernatant was discarded. The pellet was resuspended with cold 0.9% NaCl solution and centrifuged again (4000 rpm, 20 min, 4 °C). The supernatant was discarded and the pellets were frozen until the purification was performed.

Purification of OYE2 and variants

The pellets obtained from the overexpression were all treated as follows. Lysis buffer (Tris-HCl buffer, 50 mM, pH 7.5) was added to resuspend the pellet. The cells were then ultrasonicated (40% amplitude, 1 s pulse on, 4 s pulse off, total pulse time: 5 min), centrifuged (14000 rpm, 30 min, 4 °C). The supernatant was filtered through a 0.45 µM syringe filter and transferred into a 50 mL Sarstedt tube. Purification was performed by Ni-affinity chromatography: wash buffer: Tris-HCl (50 mM, 25 mM imidazole and 300 mM NaCl), elution buffer: TrisHCl (50 mM, 250 mM imidazole, 300 mM NaCl).

Bioreduction of *N*-phenyl-2-methylmaleimide

N-Phenyl-2-methylmaleimide was converted and analyzed according to published protocol.[3] Variants OYE2-H192A and Y197F were tested under standard conditions and following conversion levels were obtained: OYE2-wt: >99%; OYE2-H192A: 95%; OYE2-Y197F: 85%.

References

[1] K. Durchschein, S. Wallner, P. Macheroux, K. Zangger, W. M. F. Fabian, K. Faber, *ChemBioChem* **2012**, *13*, 2346-2351.

[2] a) M. Tiecco, L. Testaferri, M. Tingoli, D. Bartoli, *Tetrahedron* **1990**, *46*, 7139-7150; b) G. Asensio, M. A. Miranda, M. J. Sabater, J. Pérez-Prieto, A. Simón-Fuentes, R. Mello, *J. Org. Chem.* **2000**, *65*, 964-968.

[3] M. Hall, C. Stueckler, B. Hauer, R. Stuermer, T. Friedrich, M. Breuer, W. Kroutil, K. Faber, *Eur. J. Org. Chem.* **2008**, 1511-1516.
